# Narcissistic traits in young people: understanding the role of parenting and maltreatment

**DOI:** 10.1186/s40479-020-00125-7

**Published:** 2020-05-12

**Authors:** Charlotte C. van Schie, Heidi L. Jarman, Elizabeth Huxley, Brin F. S. Grenyer

**Affiliations:** 1 Illawarra Health and Medical Research Institute and the School of Psychology, University of Wollongong, Northfields Avenue, Wollongong, NSW 2522 Australia; 2 Birchtree Centre of Excellence, 58 Parramatta Road, Forest Lodge, NSW 2037 Australia

**Keywords:** Narcissism, Young people, Care, Overvaluation, Leniency, Overprotection, Overparenting, Mother and father parenting, Child maltreatment

## Abstract

**Background:**

Elevated narcissism in young people often sets up a cascade of interpersonal and mental health challenges, reinforcing the need to understand its concomitants. Experiences of maltreatment and different parenting styles have been implicated but findings to date are inconclusive. By simultaneously considering multiple remembered parenting styles and maltreatment in a large sample, this study aims to elucidate possible prognostic factors associated with both grandiose and vulnerable narcissistic traits in youth.

**Methods:**

Young people (*N* = 328, age range: 17–25 years) reported on the remembered interpersonal environment and current grandiose and vulnerable narcissism traits. Structural equation modelling was used to examine maternal and paternal parenting styles and examine the association between experiences of parenting and grandiose and vulnerable narcissism.

**Results:**

Remembered overprotection from mothers and fathers was associated with both vulnerable and grandiose narcissistic traits. Remembered maternal overvaluation related to current grandiosity, and maternal leniency related to vulnerable narcissistic traits. For paternal parenting, the combination of overvaluation and leniency and overvaluation and care explained grandiose and vulnerable traits. There was no direct effect of remembered parental care or childhood maltreatment on current levels of narcissistic traits.

**Conclusions:**

Remembered childhood experiences of being overprotected, overvalued and experiencing leniency in parental discipline, were associated with higher traits of pathological narcissism in young people. Care and maltreatment were non-specific risk factors. Remembered childhood environments of being excessively pampered are associated with grandiose and vulnerable narcissistic traits, characterised by the young person expressing unrealistic self-views, entitlement beliefs and impaired autonomy. In treatment these traits may emerge in the patient-therapist relationship and working through their developmental origins may contribute to outcomes.

## Background

Narcissistic personality disorder (NPD) and pathological narcissism are related to a high burden for both self and others [1–3]. In young people, traits of narcissism can be adaptive but when narcissism becomes pathological, it can contribute to depression, anxiety, low self-worth, suicide attempts and poor quality relationships [4–7]. Moreover, elevated narcissism during adolescence may complicate identity development for which adolescence is a formative period [8–11].

People with higher levels of pathological narcissism may have positive self-views that are not substantiated by social reality (grandiose self-view) and feelings of distress in not living up to this self-view (vulnerable self-view) [12, 13]. At the core of pathological narcissism is being unable to rely on the self and on others to maintain positive yet realistic self-esteem and self-views [10]. Despite the costs of maintaining these maladaptive self-views, they are not easily modified to a more realistic self-view [1, 14]. Factors associated with the development of narcissism are of significant interest to clinicians and researchers.

Various theories and studies implicate the role of early childhood experiences in the development of narcissism, however empirical findings are mixed. Moreover, as personality pathology can often already be observed in adolescence and tends to persist into adulthood, it is important to study pathological narcissism and potential underlying mechanisms in young people [8, 15]. Empirical examinations of narcissism and childhood experiences have examined a range of parenting behaviours including maltreatment, care, overprotection and overvaluation, and leniency.

Cold and indifferent parenting may hamper the development of an adaptive self-view [16]. It has been postulated that a lack of mirroring through cold parenting could contribute to the child’s failure to master a normal developmental process whereby a grandiose self is replaced with a more realistic view of the self [17]. However, too much mirroring through being overly sensitive to a child’s need (e.g. overparenting or pampering), is thought to be problematic as well [17, 18]. Overparenting and very lenient parenting limits the ability to learn from experiences to correct a grandiose self and may make people more reliant on others for feedback and guidance (e.g. [18–21]).

Other theorists hold that a grandiose self is developed through overvaluation by parents [22, 23]. Parental overvaluation may foster overly positive self-views, which ultimately leads to feelings of inferiority when the person interacts beyond the family system and finds the grandiose self is not supported. Moreover, combinations of parenting styles may also be associated with the development of narcissism. Freud (1914/1957) proposed that parental overvaluation, together with a lack of warmth for the child’s needs, is associated with higher traits of narcissism. Entitlement, a core aspect of both grandiose and vulnerable narcissism, may be encouraged by a parenting style that is both overvaluing and lenient [18, 23, 24].

Interpretation of current literature is complicated by a large number of mixed findings. For example, lack of parental warmth has been associated with grandiose [25–27] and vulnerable narcissism traits [25, 28]. However, others have revealed no association with grandiose [28, 29] or vulnerable narcissism [27] or, some have found higher levels of parental care with grandiose narcissism [30]. In a study examining maternal and paternal care across three cultural groups (China, Japan, and USA), low maternal care was associated with grandiose narcissism while paternal care was unrelated [31]. Grandiose narcissism appears to be associated with both general and specific mother and father effects of overvaluation [25, 29, 32]. Overparenting, overinvolvement in the child’s life to protect the child from harm and ensure certain achievements, has been related to a greater sense of entitlement and narcissism in general [33–35]. Lenient parenting has been found to relate to entitlement [21]; the opposite behaviour - greater monitoring in the form of enforcing rules - may alternatively be protective against grandiosity [30]. Conversely, another study did not find a relation between over-permissiveness and grandiose narcissism or entitlement [36]. The differences in findings could stem from what aspect of narcissism was measured (e.g. vulnerable/grandiose/total), cultural differences in expression [37], and whether maternal and paternal parenting styles were distinguished. Moreover, results may differ depending on whether childhood maltreatment was taken into account, which is a risk factor for NPD [38–40].

To summarize, overparenting, lack of warmth, leniency, overvaluation and childhood maltreatment have all been associated with higher levels of narcissism. However, these parenting behaviours have often been examined in isolation or in different combinations, with mixed findings. The current study seeks to further the understanding of grandiose and vulnerable narcissistic traits in young people and their association with a spectrum of childhood experiences of both parenting styles and maltreatment experiences. From a clinical perspective, understanding these relationships may aid in the provision of effective and timely interventions [15].

This study has three key aims. First, this study aims to build on previous research by examining remembered parenting practices and childhood maltreatment together to represent the kind of parenting environment the person experienced. Second, this study aims to extend previous research by examining parenting styles, their interactions, and their association with grandiose and vulnerable narcissistic traits. Although described in theory, the interactions between lack of warmth and overvaluation, or between leniency and overvaluation has, to the best of our knowledge, not been tested. Finally, the study aims to examine the role of parenting by mother and father figures, as some theorists have highlighted the role of the mother figure but research also indicates a role for the father figure in the development of narcissism [27, 29].

## Methods

### Participants and procedure

Participants (*N* = 328, 77% women) in this study were young people aged 17–25 (*M* = 19.28 years, *SD* = 1.63), see Table 1. A snowball method of recruitment was used, where notices for the study were provided to young people who had finished high school where those participating were further encouraged to let others know about the study. We used the definition of young people as up to the age 25 to define our sample [41]. Most participants were born in Australia (*N* = 291, 89%) and came from a family where parents were not divorced, separated or widowed (*N* = 266, 81%), see Table 1. Participants reported a broad range of trait self-esteem as measured by the Rosenberg Self-esteem Scale (RSES; *M* = 30.46, *SD* = 5.8, *Range* = 10–40) [42]. Some participants reported having been diagnosed in their life with a mental health condition (*N* = 35, 11%) with depression and anxiety as the most commonly reported diagnoses.

**Table 1:** Demographics of the sample (*N* = 328)

Demographic	N (%)/M (SD)
Gender	
Female	252 (76.8%)
Male	76 (23.2%)
Age	*M* = 19.28 (SD = 1.63)
Education	
Completed high school	318 (97%)
Completed Vocational college or training	10 (3%)
Marital status participant	
Never married	306 (93.3%)
Married	3 (0.9%)
Widowed	1 (0.3%)
Divorced or separated	18 (5.5%)
Living together	0 (0%)
Family situation parents	
Separated	8 (2.4%)
Divorced	46 (14%)
Widowed	8 (2.4%)
Not separated, divorced or widowed	266 (81.1%)
Lifetime diagnosis	35 (10.7%)
Trait self-esteem	*M* = 30.46 (SD = 5.8)

The study received ethical approval from the Institutional Review Board (HE10/370). Participants provided informed consent prior to participating. Participants completed an online assessment module via a secure website. One participant was excluded from analyses due to insincere responding. Some participants indicated not having a mother Fig. (*N* = 3), father Fig. (*N* = 17) or both (*N* = 1) and their responses could not be analysed, resulting in the sample described above (*N* = 328). Participants without mother and/or father Fig. (*N* = 21) did not differ from the rest of the sample in age, gender, education, and grandiose and vulnerable narcissism.

### Measures

#### Pathological narcissism inventory

Grandiose and vulnerable narcissism were measured using the Pathological Narcissism Inventory (PNI;13). The PNI contains 52-items that are rated on a 6-point Likert scale ranging from not at all like me (0) to very much like me [5]. Psychometric qualities of this instrument have been established [13, 43]. In this study, grandiose narcissism (GN: α = 0.87), is indicated by 18 items of the three subscales grandiose fantasy, exploitativeness and self-sacrificing self-enhancement [43]. Vulnerable narcissism (VN: α = 0.94) is indicated by 34 items of the four subscales contingent self-esteem, hiding the self, devaluing and entitlement rage [43].

#### Parental bonding instrument

Maternal and paternal parenting styles were measured using the 25-item Parental Bonding Instrument (PBI [44];), which is a widely used and extensively validated retrospective self-report measure of the bond between parent and child during the first 16 years of life [45]. Items are rated separately for the mother and father figure on a 4-point Likert scale ranging from very unlike (0) to very like [3] which form three subscales: care, overprotection and authoritarianism [46–48]. The 12-item Care scale is defined by emotional warmth, acceptance and empathy at one end and emotional coldness and rejection at the other (Maternal Care: α = 0.94, Paternal Care: α = 0.94). The 6-item Overprotection scale measures intrusiveness and risk aversion (Maternal Overprotection: α = 0.82, Paternal Overprotection: α = 0.80). Finally, the 7-item authoritarianism scale measures how much freedom was given by the parent (Maternal Authoritarianism: α = 0.81, Paternal Authoritarianism: α = 0.82). Higher scores on the authoritarianism subscale indicated more parental freedom, we therefore refer to this subscale as leniency. All three subscales showed good internal consistency for the mother and father figure.

#### Parental overvaluation

Parental overvaluation was measured by four-items used in previous studies [25, 32]. These items assessed recollections of parental overvaluation as a child that were rated on a 7-point Likert scale from strongly disagree [1] to strongly agree [7]. These items were administered separately for the mother and father figure. The scale demonstrated acceptable internal consistency (Maternal overvaluation: α = 0.68, Paternal overvaluation: α = 0.72).

#### Childhood trauma questionnaire

Experiences of maltreatment including abuse and neglect were measured using the 25-item Childhood Trauma Questionnaire (CTQ [49];). Items were rated on a 5-point Likert scale ranging from never true [1] to very often true [5]. Five subscales are comprised of five items each measuring Emotional Abuse (EA: α = 0.85), Emotional Neglect (EN: α = 0.91), Physical Abuse (PA: α = 0.84), Physical Neglect (PN: α = 0.59), and Sexual Abuse (SA: α = 0.98).

#### Statistical analyses

**Fig. 1:**
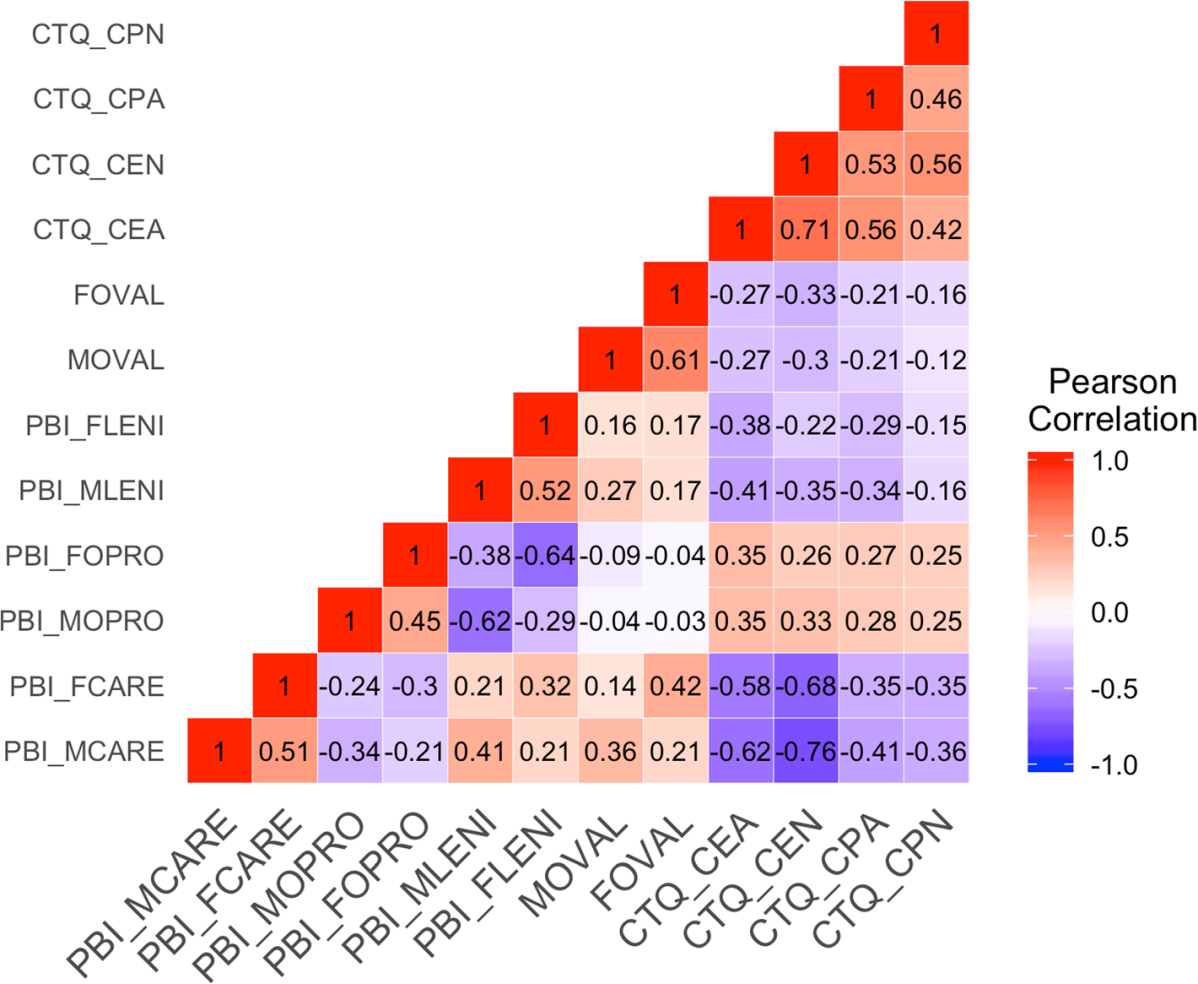
Bivariate correlations between predictor variables. Legend Fig. 1: Abbreviations: PBI = parental bonding instrument, CTQ = childhood trauma questionnaire, M = Mother figure, F = Father figure, OPRO = Overprotection, LENI = leniency, OVAL = overvaluation, CEA = childhood emotional abuse, CEN = childhood emotional neglect, CPA = childhood physical abuse, CPN = childhood physical neglect, CSA = childhood sexual abuse

Structural Equation Modelling (SEM) was used to simultaneously estimate the effect of both maternal and paternal parenting styles on grandiose and vulnerable narcissism while accounting for childhood maltreatment. First, data were checked for non-normality and multicollinearity, see Table 2, Fig. 1 and Supplemental Information. Next, a measurement model of narcissism was tested in which grandiose and vulnerable narcissism were defined as correlated latent traits indicated by the items of their respective subscales according to the model of Wright, Lukowitsky [43]. Finally, the full structural model of maternal and paternal parenting styles and childhood maltreatment relating to grandiose and vulnerable narcissism was tested, see Supplementary Fig. 1. All maternal and paternal parenting styles were allowed to covary.

**Table 2:** Means and distribution of dependent and independent observed variables. M = mother figure, F = father Fig. (N = 328)

Variable	*M* (*SD*)	Skewness	Kurtosis
PNI: Grandiose Narcissism	2.79 (SD = 0.74)	−0.08	−0.29
PNI: Vulnerable Narcissism	2.22 (SD = 0.81)	− 0.06	−0.38
PBI: Care-M	2.42 (SD = 0.63)	−1.41	1.43
PBI: Care-F	2.12 (SD = 0.71)	−0.81	−0.10
PBI: Overprotection-M	0.98 (SD = 0.67)	0.58	−0.32
PBI: Overprotection-F	0.75 (SD = 0.61)	0.80	0.09
PBI: Leniency-M	1.91 (SD = 0.59)	−0.75	0.54
PBI: Leniency-F	1.96 (SD = 0.59)	−0.64	0.55
Overvaluation-M	4.08 (SD = 1.18)	0.00	−0.18
Overvaluation-F	3.94 (SD = 1.26)	0.00	−0.20
CTQ: Emotional neglect	8.39 (SD = 4.09)	1.33	1.02
CTQ: Emotional abuse	8.21 (SD = 3.83)	1.80	3.35
CTQ: Physical neglect	6.13 (SD = 1.97)	2.50	8.04
CTQ: Physical abuse	6.32 (SD = 2.74)	3.18	11.93
CTQ: Sexual abuse	5.27 (SD = 2.04)	8.71	78.65

To ensure sufficient power to estimate all the parameters we used item parcellation for the measurement model of grandiose and vulnerable narcissism. Item parcellation is a commonly used technique and has been shown to reduce measurement error thereby increasing power, while providing a good estimate of the latent traits [50]. The items of the grandiose subscales (GF, SSSE and EXP) were divided in three parcels with six items each. Items were divided based on the item-total correlation, whereby higher and lower item-total correlations were evenly distributed over the three parcels. The item scores were averaged per parcel and parcels were used as indicators for grandiose narcissism. The same procedure was applied to the vulnerable subscales (CSE, HS, DEV and ER) whose items were divided into three parcels with 12, 11 and 11 items respectively.

In addition, we were interested in whether the difference between reported maternal and paternal parenting styles was predictive of grandiose and vulnerable narcissism. To this end, we used an intercept/slope model whereby we calculated the mean of both maternal and paternal parenting for each of the four parenting styles (warmth, overprotection, leniency and overvaluation) (intercept). We then subtracted the mean parenting style from the maternal parenting style (slope). This model tests whether the differences between maternal and paternal parenting style (slope) predict narcissism on top of how much this parenting style is present overall (intercept).

Analyses were performed with R (version 3.6.0) in RStudio (version 1.1.447). The Lavaan package (version 0.6–3) was used to perform SEM with MLR estimator, i.e. maximum likelihood estimation with robust (Huber-White) standard errors [51]. Acknowledging that cut-offs may vary depending on model complexity and sample size, a good fit for the models was evaluated using the robust CFI (> .90), scaled NFI (>.90) and scaled RMSEA (<.10) [52–54]. The chi-square is reported though may be less informative with this large sample size.

## Results

### Measurement model of PNI with parcels

The measurement model of narcissism showed a good fit to the data according to the CFI and NFI (CFI = 0.983, NFI = 0.979, RMSEA = 0.111 (CI: 0.078–0.146), scaled χ2 (8) = 40.1, *p* < .001). The large RMSEA may be due to the relatively small degrees of freedom and remaining residuals between some vulnerable item parcels and grandiose narcissism [55]. However, the CFI and NFI support this model that is in line with theory. All parcels were significantly and relatively equal indicators of the latent constructs grandiose and vulnerable narcissism. There was strong positive correlation between grandiose and vulnerable narcissism (*r* = 0.68). For all model parameters, see Supplementary Table 1.

### Parenting styles and narcissism

**Fig. 2:**
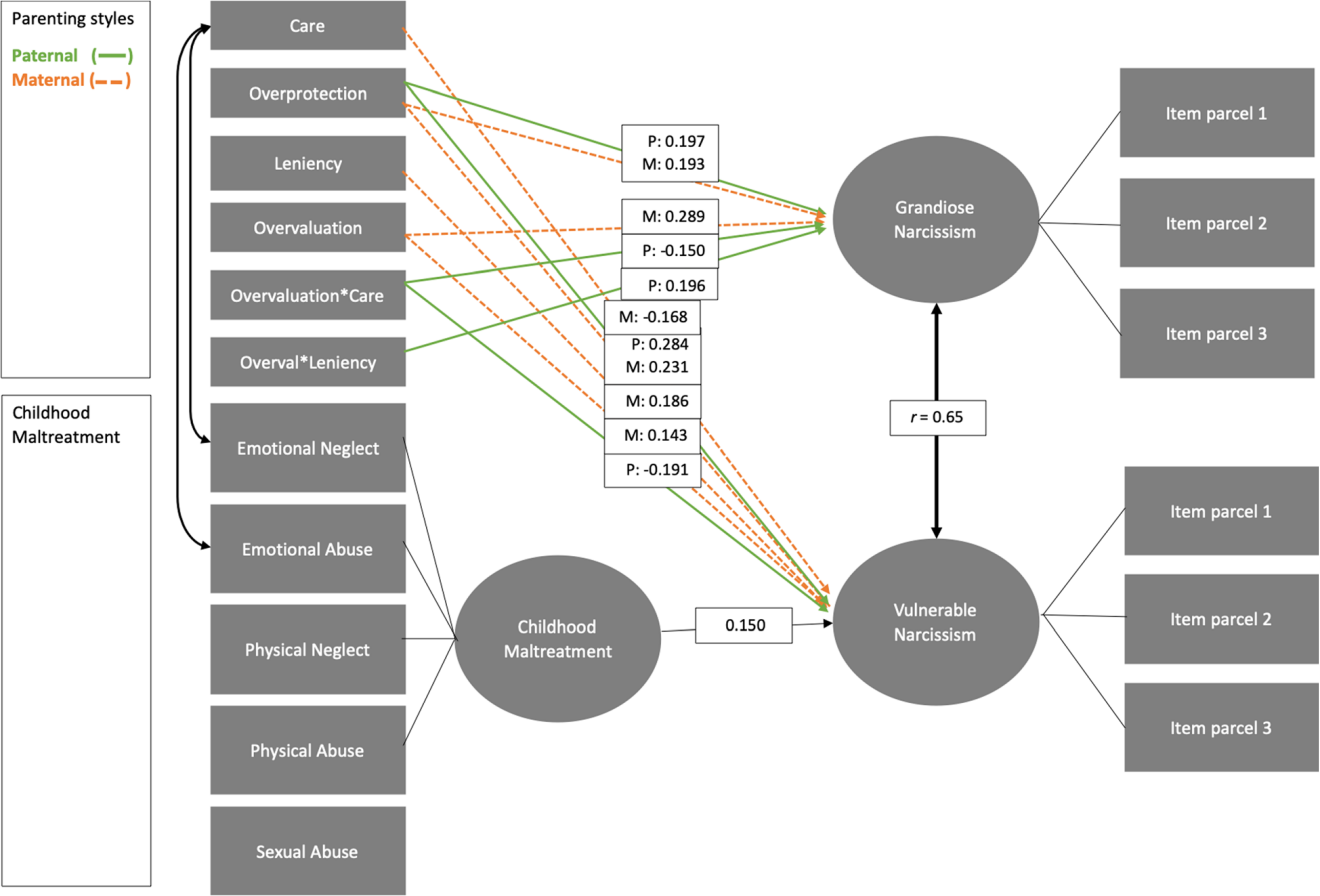
Interaction model of parenting styles and childhood maltreatment as predictors of grandiose and vulnerable narcissism. Legend Fig. 2: Model indicating significant paths only with standardized parameter estimates of regressions. Grandiose and vulnerable narcissism are indicated by three parcels each. Items are divided in parcels as follows; GN-parcel 1: 22, 23, 26, 35, 43, 45; GN-parcel 2: 1, 6, 10, 25, 42, 49; GN-parcel 3: 4, 14, 15, 31, 33, 39; VN-parcel 1: 2, 5, 11, 13, 16, 21, 27, 29, 36, 46, 47, 51; VN-parcel 2: 3, 7, 9, 12, 18, 19, 28, 30, 34, 37, 52; VN-parcel 3: 8, 17, 20, 24, 32, 38, 40, 41, 44, 48, 50

**Fig. 3:**
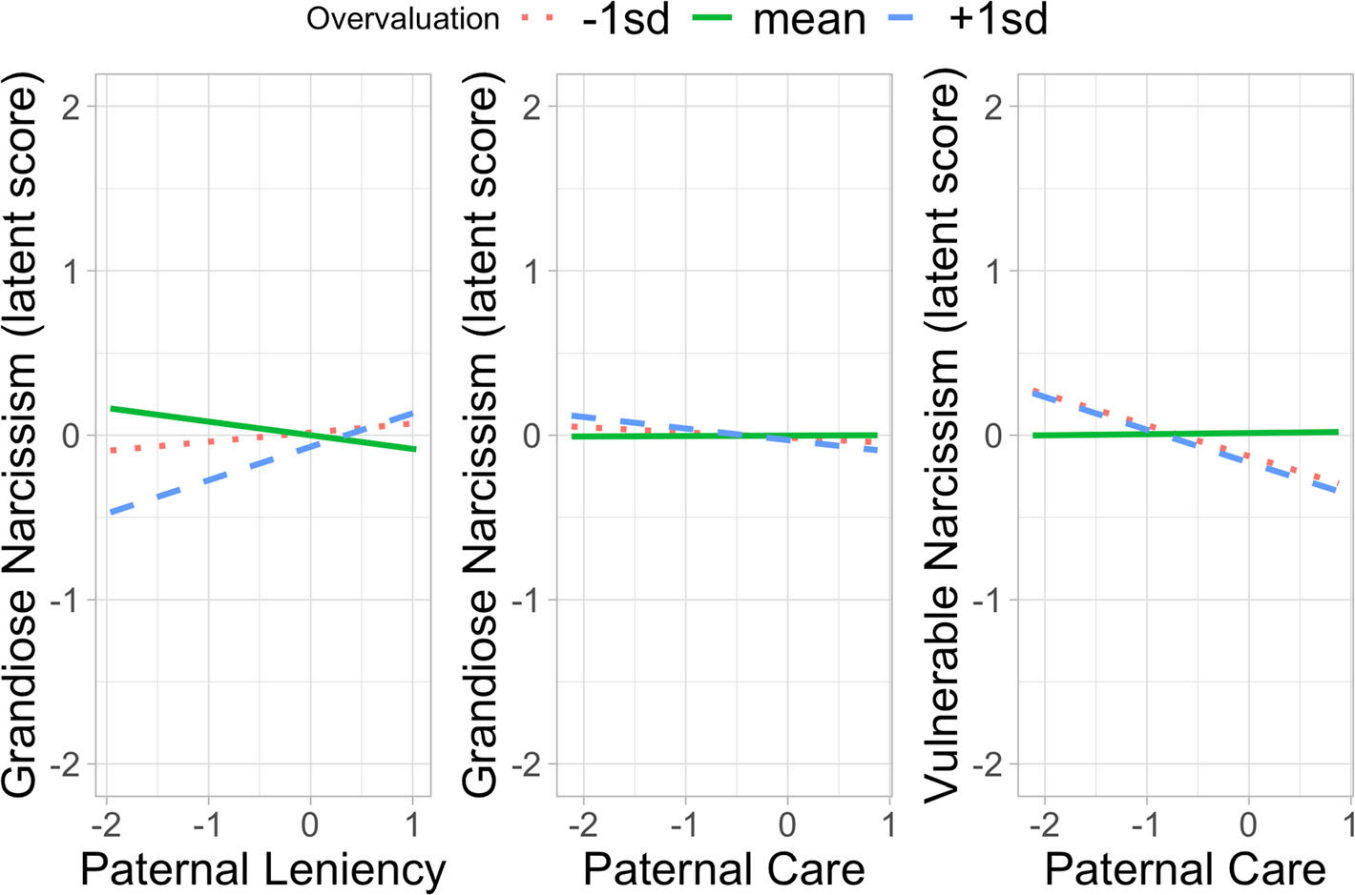
Interaction between paternal leniency and overvaluation and paternal care and overvaluation on grandiose and vulnerable narcissism

The full structural model showed a good fit to the data (CFI = 0.915, NFI = 0.877, RMSEA = 0.085 (CI: 0.077–0.093), χ2 (145) = 488.32, *p* < .001). Maternal and paternal parenting styles showed different patterns of association with grandiose and vulnerable narcissism, see Fig. 2 and for all model parameters Supplementary Table 2. Grandiose narcissism was positively associated with both maternal (*b* = 0.20, *SE* = 0.07, *p* = .007) and paternal (*b* = 0.22, *SE* = 0.08, *p* = .008) overprotection. Maternal overvaluation (*b* = 0.17, *SE* = 0.05, *p* = .001) related to higher grandiose narcissism, whereas paternal overvaluation related to grandiose narcissism only in the interaction with paternal care (*b* = − 0.10, *SE* = 0.05, *p* = .034) and paternal leniency (*b* = 0.15, *SE* = 0.05, *p* = .004). The latter interaction indicated that more overvaluation together with very lenient parenting is associated with higher grandiose narcissism, see Fig. 3. The former interaction indicated that paternal overvaluation together with a lower paternal care was associated with higher grandiose narcissism. Finally, neither care nor childhood maltreatment were associated with grandiose narcissism.

Vulnerable narcissism was positively associated with both maternal (*b* = 0.27, *SE* = 0.08, *p* < .001) and paternal (*b* = 0.37, *SE* = 0.09, *p* < .001) overprotection. In addition, maternal leniency (*b* = 0.25, *SE* = 0.10, *p* = .011) and maternal overvaluation (*b* = 0.10, *SE* = 0.05, *p* = .048) were positively associated with vulnerable narcissism. The interaction between paternal care and overvaluation (*b* = − 0.14, *SE* = 0.05, *p* = 0.007) significantly predicted vulnerable narcissism, see Fig. 3. This interaction indicates that remembered paternal care influences the association between overvaluation and grandiose and vulnerable narcissism. Finally, maternal care (*b* = − 0.24, *SE* = 0.11, *p* = 0.030) and childhood maltreatment (*b* = 0.04, *SE* = 0.02, *p* = 0.028) were significantly related to vulnerable narcissism.

### Differences between maternal and paternal parenting

To examine whether the difference between maternal and paternal parenting style was associated with grandiose and vulnerable narcissism, an intercept-slope model was tested combining the average parenting style (intercept) with the distance between the maternal and average score (slope). This model demonstrated a good fit (CFI = 0.935, NFI = 0.900, RMSEA = 0.072 (CI: 0.063–0.081), χ2 (128) = 345.51, *p* < .001). The difference between maternal and paternal overvaluation (*b* = 0.18, *SE* = 0.09, *p* = .047) was related to grandiose narcissism, with higher scores for the mother figure compared to the father figure relating to higher grandiose narcissism. No other differences between maternal and paternal parenting were related to narcissism, see Supplementary Table 3 for all parameter estimates.

## Discussion

In this study, we simultaneously investigated how maternal and paternal parenting and maltreatment experiences relate to grandiose and vulnerable narcissism in young people.

### Overprotection as the common denominator

Maternal and paternal parenting styles demonstrated different patterns of association with grandiose and vulnerable narcissism. However, one striking commonality was the association between maternal and paternal overprotection and grandiose and vulnerable narcissism. Although overprotection as measured by the PBI has not been studied in relation to pathological narcissism, similar concepts such as overparenting and ‘helicopter parenting’ where parents are overinvolved in a child’s life, have been linked to a greater sense of entitlement and pathological narcissism in general [33–35]. This study indicated that even when taking memories of other parenting styles such as care and overvaluation into account, overprotection by either mother or father is associated with elevated narcissism. Overprotection may limit the ability to learn from one’s own experiences and make people less autonomous, i.e. more reliant on others for feedback and guidance (e.g. 18, 19, 20). Indeed, overparenting is related to lower self-efficacy and coping skills, particularly in young people [34, 35, 56, 57]. Moreover, vulnerable narcissism is related to more negative self-beliefs regarding autonomy [58]. In sum, overprotective parenting may limit the learning experiences for children and young adults, which may foster entitlement and negative self-beliefs about impaired autonomy, which in turn may make individuals more prone to develop elevated narcissistic traits.

### Overvaluation and grandiose narcissism

Remembered parental overvaluation was strongly associated with grandiose narcissism. The association between overvaluation and narcissism is highlighted by psychodynamic and social-learning theories [22, 23, 59]. Overvaluation has been proposed to directly stimulate unrealistic positive self-views [22, 23]. Freud explained this as parents who ascribe every perfection to their child even when sober observation would find no occasion to do so (59, pp. 90–91). Research has indicated that children, who are praised regardless of achievement or effort, become more afraid of failing and use avoidance and cheating tactics to maintain a positive self-view [60–62]. This hypervigilance to threats to the self and defensive reactions have also been observed in narcissism [63–67].

Our findings support the idea that a grandiose self may be explicitly fostered by parental figures through unconditional praise, and therefore a lack of care may not be sufficient to explain grandiose self-views [16, 17]. Another study also found that overvaluation as opposed to warmth predicted narcissism [29]. However, it should be noted that we found that paternal care was a protective factor for paternal overvaluation in the relation to grandiose as well as vulnerable narcissism. Greater paternal care may indicate that in addition to overvaluation there is attention to the child’s needs. Invalidating a child’s needs has been related to higher grandiose and vulnerable narcissism [27, 68]. It may thus be the combination of paternal overvaluation with lack of care for the child’s needs, that is important in the development of elevated narcissism [59].

### Leniency and vulnerable narcissism

Remembered maternal leniency was associated only with vulnerable narcissism. Similar to overparenting, lenient parenting is thought to limit the ability to learn from own experiences albeit through too much freedom as opposed to restrictions in exploration [18, 20, 21, 69]. Lack of limit setting may have consequences for developing a sense of reality and self-discipline [69] which may be expressed as entitlement rage relevant to vulnerable narcissism [14, 43, 69]. Specifically, a sense of entitlement, which is relevant to both grandiose and vulnerable narcissism, may be fostered by the combination of overvaluing and lenient parenting, i.e. parents who praise a child and do not set many boundaries [18, 21, 23, 24, 58]. Interestingly, our findings indicate that paternal leniency and overvaluation were associated with grandiose narcissism.

### Differences in maternal and paternal parenting

Overall, the findings suggest that remembered maternal parenting has a stronger association with narcissism whereas for paternal parenting the combination of parenting styles was relevant. Other studies have also found differences in maternal and paternal parenting with a stronger association for maternal parenting [21, 27]. Several factors should be considered in explaining this finding. First, the mother figure may often be the primary caregiver and as such, more direct effects are observed for maternal parenting and more indirect effects of paternal parenting [70]. However, it may also differ per parenting style as overprotection had a direct effect for both the mother and father figure whereas overvaluation and leniency had differential effects. Second, there could be different expectations, norms and needs regarding the parenting role of the mother and father fig [71, 72]. Whereas maternal and paternal parenting are conceptually the same, studies suggest that mothers are often more involved in all parenting domains than fathers [70, 71, 73]. With the exception of overprotection, it may be the case that maternal parenting is more strongly associated with narcissism, so that paternal parenting is only associated with narcissism under certain circumstances (e.g. when overvaluation is accompanied by low care or high leniency).

### Parental care and childhood maltreatment

We found that remembered maternal care or childhood maltreatment related to the presence of traits of vulnerable but not grandiose narcissism. Findings for parental care and childhood maltreatment in relation to narcissism have been mixed in previous studies (see e.g. [25, 27, 29, 74–76]). The role of care and childhood maltreatment may therefore be more nuanced. In this study, other parenting behaviours seem more strongly related to narcissism than care or childhood maltreatment. However, the interaction effects with paternal care may indicate that care is a protective factor in grandiose and vulnerable narcissism. Note however that findings suggest that although child maltreatment has been found to be a risk factor in the development of narcissistic personality disorder (NPD), it is also a risk factor for other (personality) disorders [38–40]. It could be thought that care and childhood maltreatment are probably protective and risk factors respectively but not necessarily specific contributors to the development of narcissism.

### Implications and future research

Remembered parental overprotection, overvaluation, leniency and to a lesser extent, care played an important role in explaining the presence of traits of grandiose and vulnerable narcissism. Through overvaluation, self-views may become overly positive and not grounded in reality. Through overprotection or leniency, these self-views may not be corrected as there are less opportunities to learn from own experiences (overprotection) or learn realistic restrictions (leniency). Under these conditions, the opportunity to learn a more adaptive self-view is further inhibited [77]. Moreover, maladaptive self-views may negatively impact interactions with others, such as becoming defensively aggressive [7, 66, 67, 78]. Our findings regarding overvaluation and narcissism suggest that praise is proportionate to achievement or effort to encourage adaptive self-views [79]. With respect to overprotection and leniency, children need safe opportunities to explore i.e. being given the freedom to explore within a set of boundaries as to foster a sense of autonomy and self-discipline. Future research should further investigate the exact mechanisms by which certain (combinations of) parenting styles lead to the development of specific characteristics (e.g., autonomy, self-discipline, adaptive self-views) that may be related to elevated narcissism, preferably using longitudinal designs.

### Strengths and limitations

Parenting behaviours have traditionally been examined in isolation. We simultaneously examined maternal and paternal parenting styles and maltreatment and their relation to narcissism. Moreover, by distinguishing between grandiose and vulnerable narcissism, we were able to examine more specific relations between parenting styles and different aspects of narcissism.

The current study also has a number of limitations. The use of retrospective self-report measures of parenting practices is limited by shared method variances and, as this is a cross-sectional study, no causality can be inferred. The association between narcissism and parenting may also be influenced by participant’s current self-perception. In particular, the social cognitive effects of narcissism, which influence memory recall, perception and attentional biases, may influence individual’s recall of their childhood experiences. Future studies should aim to examine parenting styles and narcissism using longitudinal study designs, and other-report assessment of parenting styles. Moreover, we could not compare narcissism to different classes of psychopathology and findings may therefore be indicative of psychopathology in general. Finally, as our sample was predominantly female, we could not perform analyses on gender differences in the sample. With larger male samples this may be further explored.

## Conclusions

Considering multiple parenting styles and maltreatment, remembered parental overprotection, overvaluation and leniency appeared to be associated with higher narcissistic traits in young people. In addition to overprotection, parental overvaluation was associated with greater grandiose narcissism, and parental leniency with more vulnerable narcissism. These findings were strongest in relation to maternal parenting. Lack of paternal care and child maltreatment were non-specific risk factors for elevated narcissism. The environment a child grows up in, may be associated with the development of unrealistic self-views, entitlement and impaired autonomy observed in narcissism. These findings also have implications for treatment, not only in understanding putative developmental factors, but also the possible patient-therapist relationship challenges in the therapy process stemming from these narcissistic beliefs [80].

## Supplementary information

**Additional file 1.** Supplemental information

## Data Availability

The dataset analysed during the current study does not have clearance to be made publicly available but is available from the corresponding author on reasonable request. Moreover, covariance matrices and the analysis script used are provided on open science framework (https://osf.io/c3gv9/).
