# Supplementary material for: Narcissistic traits in young people: understanding the role of parenting and maltreatment
Source: Borderline Personal Disord Emot Dysregul. 2020 May 12;7:10. doi: 10.1186/s40479-020-00125-7 (PMC7216544; doi:10.1186/s40479-020-00125-7)
Supplement: Supplementary file 1 — Additional file 1. Supplemental information [file 40479_2020_125_MOESM1_ESM.docx]

# Supplemental information

#### Exploratory data analysis

Skewness and kurtosis were checked for the predictor and outcome variables, see Table 2. Childhood sexual abuse and to a lesser degree childhood physical abuse showed deviations in skewness (> 3) and kurtosis (> 10) (1). To deal with the non-normality in the data when performing SEM, we used the maximum likelihood estimator with robust standard errors and scaled statistics (MLR) (1, 2). Bivariate correlations between predictors of parenting style and childhood maltreatment subscales indicated that there was potential multicollinearity between maternal and paternal warmth on the one hand and childhood emotional abuse and neglect on the other hand, see Figure 1. These strong relations were captured by allowing maternal and paternal warmth on the one hand and childhood emotional abuse and neglect on the other hand to covary.

*SEM data analysis*

For the full structural model, the parenting styles are represented by the PBI subscales and the overvaluation scale. Childhood maltreatment is represented as a latent variable indicated by the CTQ subscales. To calculate the interaction effects of warmth by overvaluation and leniency by overvaluation, the subscales of the PBI and the overvaluation scale were mean centered for mother and father figure separately. The interaction was then calculated as a product of the centered scores of warmth by overvaluation and leniency by overvaluation resulting in two interaction effects for maternal and paternal parenting. The centered variables were used in the SEM analysis.

*Measurement model of childhood maltreatment*

The measurement model of childhood maltreatment in which all five CTQ subscales load on one latent variable, showed a relatively good fit to the data (CFI = 0.949, NFI = 0.941, RMSEA = 0.128 (CI: 0.088-0.172), scaled χ2 (5) = 31.9, p < .001). The RMSEA may be large due to the small number of degrees of freedom (3).

*Supplementary figures*


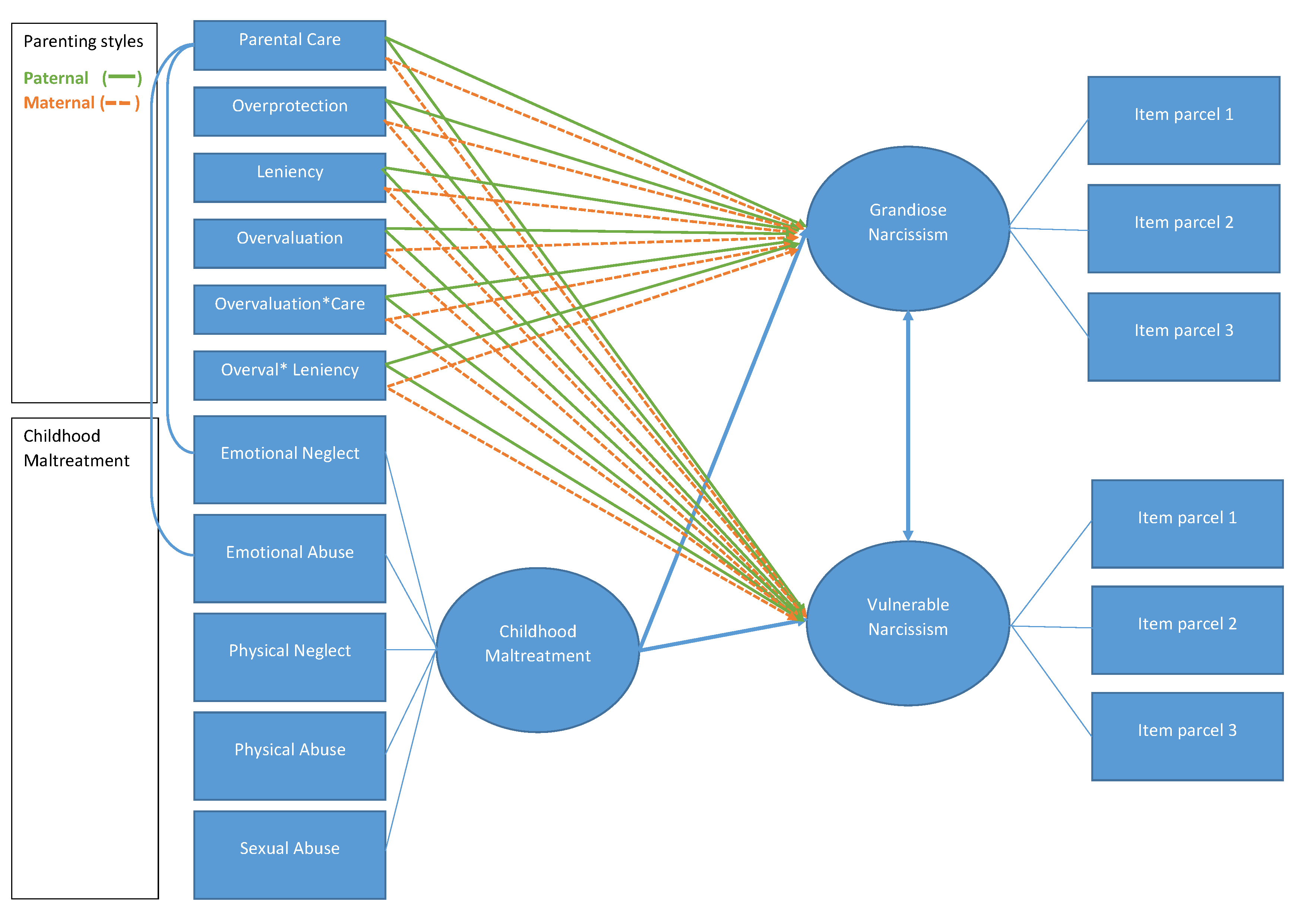


Figure S 1. Full structural model tested of parenting styles and childhood maltreatment as predictors of grandiose (GN) and vulnerable (VN) narcissism indicated by three parcels each. Items are divided in parcels as follows; GN-parcel 1: 22, 23, 26, 35, 43, 45; GN-parcel 2: 1, 6, 10, 25, 42, 49; GN-parcel 3: 4, 14, 15, 31, 33, 39; VN-parcel 1: 2, 5, 11, 13, 16, 21, 27, 29, 36, 46, 47, 51; VN-parcel 2: 3, 7, 9, 12, 18, 19, 28, 30, 34, 37, 52; VN-parcel 3: 8, 17, 20, 24, 32, 38, 40, 41, 44, 48, 50. All parenting styles were allowed to covary.

Supplementary Tables

Table S 1. Parameters estimates of measurement model of narcissism. Standardized parameters are based on completely standardized solution where both latent variables and indicators are standardized.

| Latent variable | Indicator | Parameter estimate | Standard error | z-value | p- value | Standardized parameter |
| --- | --- | --- | --- | --- | --- | --- |
| Grandiose Narcissism | PNI GN parcel 1 | 1.00 | 0.00 |  |  | 0.87 |
|  | PNI GN parcel 2 | 1.04 | 0.05 | 20.66 | **< .001** | 0.87 |
|  | PNI GN parcel 3 | 1.13 | 0.05 | 21.65 | **< .001** | 0.90 |
| Vulnerable Narcissism | PNI VN parcel 1 | 1.00 | 0.00 |  |  | 0.94 |
|  | PNI VN parcel 2 | 0.98 | 0.03 | 31.40 | **< .001** | 0.93 |
|  | PNI VN parcel 3 | 1.00 | 0.03 | 31.87 | **< .001** | 0.93 |

Table S 2. Parameters estimates of final model of mother and father parenting and narcissism. Standardized parameters are based on completely standardized solution where both latent variables and indicators are standardized.

| Latent Variables | Indicator | Parameter estimate | Standard error | z-value | p- value | Standardized parameter |
| --- | --- | --- | --- | --- | --- | --- |
| Grandiose Narcissism | PNI GN parcel 1 | 1.00 | 0.00 |  |  | 0.87 |
|  | PNI GN parcel 2 | **1.04** | **0.05** | **20.64** | **<.001** | **0.87** |
|  | PNI GN parcel 3 | **1.13** | **0.06** | **19.14** | **<.001** | **0.90** |
| Vulnerable Narcissism | PNI VN parcel 1 | 1.00 | 0.00 |  |  | 0.94 |
|  | PNI VN parcel 2 | **0.98** | **0.03** | **31.98** | **<.001** | **0.93** |
|  | PNI VN parcel 3 | **1.00** | **0.03** | **31.24** | **<.001** | **0.93** |
| Childhood Trauma | CTQ EA | 1.00 | 0.00 |  |  | 0.81 |
|  | CTQ EN | **0.90** | **0.11** | **8.05** | **<.001** | **0.73** |
|  | CTQ PA | **0.57** | **0.13** | **4.40** | **<.001** | **0.60** |
|  | CTQ PN | **0.36** | **0.08** | **4.23** | **<.001** | **0.52** |
|  | CTQ SA | 0.13 | 0.09 | 1.40 | 0.162 | 0.18 |
| Regressions |  | Parameter estimate | Standard error | z-value | p- value | Standardized parameter |
| Grandiose Narcissism | PBI M CARE | -0.13 | 0.10 | -1.29 | 0.198 | -0.11 |
|  | PBI F CARE | 0.02 | 0.08 | 0.31 | 0.758 | 0.02 |
|  | PBI M OVERPRO | **0.20** | **0.07** | **2.70** | **0.007** | **0.19** |
|  | PBI F OVERPRO | **0.22** | **0.08** | **2.64** | **0.008** | **0.20** |
|  | PBI M LENIENCY | 0.07 | 0.09 | 0.82 | 0.412 | 0.06 |
|  | PBI F LENIENCY | 0.14 | 0.09 | 1.59 | 0.111 | 0.12 |
|  | M OVERVALUATION | **0.17** | **0.05** | **3.30** | **0.001** | **0.29** |
|  | F OVERVALUATION | -0.04 | 0.05 | -0.82 | 0.413 | -0.07 |
|  | M CARE*OVAL | 0.09 | 0.08 | 1.26 | 0.210 | 0.11 |
|  | F CARE*OVAL | **-0.10** | **0.05** | **-2.13** | **0.034** | **-0.15** |
|  | M OVAL*LENI | -0.10 | 0.06 | -1.52 | 0.129 | -0.11 |
|  | F OVAL*LENI | **0.15** | **0.05** | **2.90** | **0.004** | **0.20** |
|  | Childhood Trauma | 0.01 | 0.02 | 0.51 | 0.607 | 0.04 |
| Vulnerable Narcissism | PBI M CARE | **-0.24** | **0.11** | **-2.18** | **0.030** | **-0.17** |
|  | PBI F CARE | -0.08 | 0.08 | -0.92 | 0.356 | -0.06 |
|  | PBI M OVERPRO | **0.27** | **0.08** | **3.45** | **0.001** | **0.23** |
|  | PBI F OVERPRO | **0.37** | **0.09** | **4.07** | **<.001** | **0.28** |
|  | PBI M LENIENCY | **0.25** | **0.10** | **2.53** | **0.011** | **0.19** |
|  | PBI F LENIENCY | 0.05 | 0.11 | 0.43 | 0.665 | 0.03 |
|  | M OVERVALUATION | **0.10** | **0.05** | **1.98** | **0.048** | **0.14** |
|  | F OVERVALUATION | -0.02 | 0.05 | -0.34 | 0.734 | -0.03 |
|  | M CARE*OVAL | 0.08 | 0.08 | 0.99 | 0.323 | 0.09 |
|  | F CARE*OVAL | **-0.14** | **0.05** | **-2.71** | **0.007** | **-0.19** |
|  | M OVAL*LENI | -0.06 | 0.08 | -0.78 | 0.436 | -0.06 |
|  | F OVAL*LENI | 0.09 | 0.06 | 1.43 | 0.153 | 0.10 |
|  | Childhood Trauma | **0.04** | **0.02** | **2.20** | **0.028** | **0.15** |
| Covariances |  | Parameter estimate | Standard error | z-value | p- value | Standardized parameter |
| CTQ EN | PBI M CARE | -0.92 | 0.15 | -6.28 | <.001 | -0.68 |
| CTQ EA | PBI M CARE | -0.41 | 0.15 | -2.73 | 0.006 | -0.36 |
| CTQ EN | PBI F CARE | -0.97 | 0.16 | -6.23 | <.001 | -0.61 |
| CTQ EA | PBI F CARE | -0.51 | 0.15 | -3.46 | 0.001 | -0.38 |
| Grandiose Narcissism | Vulnerable Narcissism | 0.28 | 0.03 | 8.46 | <.001 | 0.65 |
| PBI M CARE | PBI F CARE | 0.14 | 0.02 | 6.38 | <.001 | 0.40 |
| PBI M CARE | PBI M OVERPRO | -0.09 | 0.02 | -3.95 | <.001 | -0.25 |
| PBI M CARE | PBI F OVERPRO | -0.05 | 0.02 | -2.92 | 0.004 | -0.14 |
| PBI M CARE | PBI M LENIENCY | 0.10 | 0.02 | 4.42 | <.001 | 0.32 |
| PBI M CARE | PBI F LENIENCY | 0.05 | 0.02 | 2.67 | 0.008 | 0.16 |
| PBI M CARE | M OVERVALUATION | 0.17 | 0.04 | 4.54 | <.001 | 0.26 |
| PBI M CARE | F OVERVALUATION | 0.05 | 0.04 | 1.28 | 0.202 | 0.08 |
| PBI M CARE | M CARE*OVAL | -0.16 | 0.05 | -3.48 | 0.001 | -0.35 |
| PBI M CARE | F CARE*OVAL | -0.12 | 0.05 | -2.25 | 0.025 | -0.20 |
| PBI M CARE | M OVAL*LENI | -0.09 | 0.03 | -3.00 | 0.003 | -0.22 |
| PBI M CARE | F OVAL*LENI | -0.05 | 0.03 | -1.52 | 0.128 | -0.10 |
| PBI F CARE | PBI M OVERPRO | -0.06 | 0.03 | -1.96 | 0.050 | -0.13 |
| PBI F CARE | PBI F OVERPRO | -0.10 | 0.03 | -3.84 | <.001 | -0.24 |
| PBI F CARE | PBI M LENIENCY | 0.03 | 0.03 | 1.20 | 0.229 | 0.09 |
| PBI F CARE | PBI F LENIENCY | 0.10 | 0.03 | 3.92 | <.001 | 0.27 |
| PBI F CARE | M OVERVALUATION | 0.01 | 0.05 | 0.19 | 0.847 | 0.01 |
| PBI F CARE | F OVERVALUATION | 0.26 | 0.05 | 4.80 | <.001 | 0.31 |
| PBI F CARE | M CARE*OVAL | -0.05 | 0.04 | -1.26 | 0.206 | -0.10 |
| PBI F CARE | F CARE*OVAL | -0.21 | 0.07 | -2.94 | 0.003 | -0.30 |
| PBI F CARE | M OVAL*LENI | -0.03 | 0.04 | -0.64 | 0.524 | -0.05 |
| PBI F CARE | F OVAL*LENI | -0.10 | 0.05 | -1.95 | 0.051 | -0.18 |
| PBI M OVERPRO | PBI F OVERPRO | 0.19 | 0.03 | 7.33 | <.001 | 0.45 |
| PBI M OVERPRO | PBI M LENIENCY | -0.24 | 0.03 | -8.34 | <.001 | -0.62 |
| PBI M OVERPRO | PBI F LENIENCY | -0.11 | 0.03 | -4.56 | <.001 | -0.29 |
| PBI M OVERPRO | M OVERVALUATION | -0.03 | 0.05 | -0.62 | 0.538 | -0.04 |
| PBI M OVERPRO | F OVERVALUATION | -0.03 | 0.05 | -0.50 | 0.618 | -0.03 |
| PBI M OVERPRO | M CARE*OVAL | 0.13 | 0.04 | 3.40 | 0.001 | 0.24 |
| PBI M OVERPRO | F CARE*OVAL | 0.11 | 0.06 | 1.84 | 0.066 | 0.15 |
| PBI M OVERPRO | M OVAL*LENI | 0.13 | 0.04 | 2.98 | 0.003 | 0.26 |
| PBI M OVERPRO | F OVAL*LENI | 0.09 | 0.05 | 1.90 | 0.058 | 0.16 |
| PBI F OVERPRO | PBI M LENIENCY | -0.14 | 0.02 | -5.95 | <.001 | -0.38 |
| PBI F OVERPRO | PBI F LENIENCY | -0.23 | 0.03 | -8.55 | <.001 | -0.64 |
| PBI F OVERPRO | M OVERVALUATION | -0.06 | 0.04 | -1.50 | 0.134 | -0.09 |
| PBI F OVERPRO | F OVERVALUATION | -0.03 | 0.05 | -0.64 | 0.525 | -0.04 |
| PBI F OVERPRO | M CARE*OVAL | 0.07 | 0.03 | 2.18 | 0.029 | 0.15 |
| PBI F OVERPRO | F CARE*OVAL | 0.12 | 0.06 | 2.01 | 0.044 | 0.19 |
| PBI F OVERPRO | M OVAL*LENI | 0.09 | 0.04 | 2.24 | 0.025 | 0.18 |
| PBI F OVERPRO | F OVAL*LENI | 0.14 | 0.05 | 2.58 | 0.010 | 0.27 |
| PBI M LENIENCY | PBI F LENIENCY | 0.18 | 0.03 | 7.08 | <.001 | 0.52 |
| PBI M LENIENCY | M OVERVALUATION | 0.18 | 0.04 | 4.42 | <.001 | 0.27 |
| PBI M LENIENCY | F OVERVALUATION | 0.13 | 0.05 | 2.72 | 0.007 | 0.17 |
| PBI M LENIENCY | M CARE*OVAL | -0.14 | 0.04 | -3.34 | 0.001 | -0.28 |
| PBI M LENIENCY | F CARE*OVAL | -0.13 | 0.06 | -2.27 | 0.023 | -0.21 |
| PBI M LENIENCY | M OVAL*LENI | -0.16 | 0.05 | -3.28 | 0.001 | -0.35 |
| PBI M LENIENCY | F OVAL*LENI | -0.11 | 0.05 | -2.33 | 0.020 | -0.21 |
| PBI F LENIENCY | M OVERVALUATION | 0.11 | 0.04 | 2.62 | 0.009 | 0.16 |
| PBI F LENIENCY | F OVERVALUATION | 0.13 | 0.05 | 2.71 | 0.007 | 0.18 |
| PBI F LENIENCY | M CARE*OVAL | -0.04 | 0.03 | -1.34 | 0.182 | -0.09 |
| PBI F LENIENCY | F CARE*OVAL | -0.14 | 0.06 | -2.20 | 0.028 | -0.22 |
| PBI F LENIENCY | M OVAL*LENI | -0.06 | 0.04 | -1.67 | 0.096 | -0.14 |
| PBI F LENIENCY | F OVAL*LENI | -0.17 | 0.06 | -2.81 | 0.005 | -0.34 |
| M OVERVAL | F OVERVALUATION | 0.90 | 0.10 | 9.49 | <.001 | 0.61 |
| M OVERVAL | M CARE*OVAL | -0.13 | 0.10 | -1.32 | 0.187 | -0.13 |
| M OVERVAL | F CARE*OVAL | -0.13 | 0.10 | -1.29 | 0.197 | -0.10 |
| M OVERVAL | M OVAL*LENI | -0.12 | 0.08 | -1.38 | 0.168 | -0.13 |
| M OVERVAL | F OVAL*LENI | -0.05 | 0.08 | -0.60 | 0.546 | -0.05 |
| F OVERVAL | M CARE*OVAL | -0.01 | 0.08 | -0.16 | 0.877 | -0.01 |
| F OVERVAL | F CARE*OVAL | -0.33 | 0.14 | -2.45 | 0.014 | -0.25 |
| F OVERVAL | M OVAL*LENI | -0.09 | 0.08 | -1.11 | 0.266 | -0.09 |
| F OVERVAL | F OVAL*LENI | -0.13 | 0.11 | -1.15 | 0.251 | -0.12 |
| M CARE*OVAL | F CARE*OVAL | 0.42 | 0.11 | 3.91 | <.001 | 0.47 |
| M CARE*OVAL | M OVAL*LENI | 0.31 | 0.09 | 3.69 | <.001 | 0.51 |
| M CARE*OVAL | F OVAL*LENI | 0.13 | 0.07 | 2.01 | 0.045 | 0.19 |
| F CARE*OVAL | M OVAL*LENI | 0.24 | 0.10 | 2.36 | 0.018 | 0.30 |
| F CARE*OVAL | F OVAL*LENI | 0.50 | 0.16 | 3.12 | 0.002 | 0.53 |
| M OVAL*LENI | F OVAL*LENI | 0.26 | 0.08 | 3.14 | 0.002 | 0.40 |

Table S 3. Parameters estimates of differences in mother and father parenting and narcissism (Intercept-slope model). Standardized parameters are based on completely standardized solution where both latent variables and indicators are standardized.

| Latent Variables | Indicator | Parameter estimate | Standard error | z-value | p- value | Standardized parameter |
| --- | --- | --- | --- | --- | --- | --- |
| Grandiose Narcissism | PNI GN parcel 1 | 1.00 | 0.00 |  |  | 0.87 |
|  | PNI GN parcel 2 | **1.04** | **0.05** | **20.60** | **<.001** | **0.87** |
|  | PNI GN parcel 3 | **1.13** | **0.06** | **19.07** | **<.001** | **0.90** |
| Vulnerable Narcissism | PNI VN parcel 1 | 1.00 | 0.00 |  |  | 0.94 |
|  | PNI VN parcel 2 | **0.98** | **0.03** | **32.02** | **<.001** | **0.93** |
|  | PNI VN parcel 3 | **1.00** | **0.03** | **31.22** | **<.001** | **0.93** |
| Childhood Trauma | CTQ EA | 1.00 | 0.00 |  |  | 0.81 |
|  | CTQ EN | **0.91** | **0.12** | **7.90** | **<.001** | **0.73** |
|  | CTQ PA | **0.56** | **0.14** | **4.16** | **<.001** | **0.58** |
|  | CTQ PN | **0.36** | **0.09** | **4.14** | **<.001** | **0.52** |
|  | CTQ SA | 0.13 | 0.09 | 1.37 | 0.171 | 0.18 |
| Regressions |  | Parameter estimate | Standard error | z-value | p- value | Standardized parameter |
| Grandiose Narcissism | PBI Mean Parental Care | -0.13 | 0.08 | -1.57 | 0.116 | -0.10 |
|  | PBI Parental diff in Care | -0.18 | 0.15 | -1.19 | 0.235 | -0.09 |
|  | PBI Mean Parental Leniency | **0.20** | **0.09** | **2.18** | **0.030** | **0.15** |
|  | PBI Parental diff in Leniency | 0.01 | 0.15 | 0.06 | 0.950 | 0.00 |
|  | PBI Mean Parental Overpro | **0.42** | **0.09** | **4.94** | **<.001** | **0.34** |
|  | PBI Parental diff in Overpro | -0.01 | 0.13 | -0.11 | 0.916 | -0.01 |
|  | Mean Parental Overvaluation | **0.14** | **0.04** | **3.55** | **<.001** | **0.23** |
|  | Parental diff in Overvaluation | **0.18** | **0.09** | **1.98** | **0.048** | **0.14** |
|  | Mean Parental Care*Oval | -0.03 | 0.07 | -0.42 | 0.677 | -0.04 |
|  | Mean Parental Oval*Leni | 0.05 | 0.07 | 0.79 | 0.431 | 0.05 |
|  | Childhood Trauma | 0.01 | 0.02 | 0.44 | 0.658 | 0.03 |
| Vulnerable Narcissism | PBI Mean Parental Care | **-0.35** | **0.10** | **-3.52** | **<.001** | **-0.22** |
|  | PBI Parental diff in Care | -0.21 | 0.16 | -1.30 | 0.194 | -0.09 |
|  | PBI Mean Parental Leniency | **0.29** | **0.10** | **2.79** | **0.005** | **0.19** |
|  | PBI Parental diff in Leniency | 0.24 | 0.18 | 1.37 | 0.170 | 0.09 |
|  | PBI Mean Parental Overpro | **0.65** | **0.10** | **6.68** | **<.001** | **0.45** |
|  | PBI Parental diff in Overpro | -0.08 | 0.14 | -0.57 | 0.569 | -0.03 |
|  | Mean Parental Overvaluation | **0.09** | **0.04** | **2.24** | **0.025** | **0.13** |
|  | Parental diff in Overvaluation | 0.08 | 0.09 | 0.90 | 0.366 | 0.05 |
|  | Mean Parental Care*Oval | -0.09 | 0.08 | -1.19 | 0.235 | -0.09 |
|  | Mean Parental Oval*Leni | 0.00 | 0.08 | -0.01 | 0.996 | 0.00 |
|  | Childhood Trauma | **0.04** | **0.02** | **2.10** | **0.036** | **0.14** |
| Covariances |  | Parameter estimate | Standard error | z-value | p- value | Standardized parameter |
| CTQ EN | PBI Mean Parental Care | -0.99 | 0.15 | -6.75 | <.001 | -0.80 |
| CTQ EA | PBI Mean Parental Care | -0.49 | 0.15 | -3.25 | 0.001 | -0.47 |
| Grandiose Narcissism | Vulnerable Narcissism | 0.29 | 0.03 | 8.57 | <.001 | 0.65 |
| PBI Mean Parental Care | PBI Parental diff in Care | -0.03 | 0.01 | -3.50 | 0.000 | -0.16 |
| PBI Mean Parental Care | PBI Mean Parental Leniency | 0.07 | 0.02 | 3.81 | 0.000 | 0.27 |
| PBI Mean Parental Care | PBI Parental diff in Leniency | -0.01 | 0.01 | -0.60 | 0.549 | -0.03 |
| PBI Mean Parental Care | PBI Mean Parental Overpro | -0.07 | 0.02 | -4.17 | 0.000 | -0.25 |
| PBI Mean Parental Care | PBI Parental diff in Overpro | 0.00 | 0.01 | -0.08 | 0.938 | 0.00 |
| PBI Mean Parental Care | Mean Parental Overvaluation | 0.12 | 0.04 | 3.31 | 0.001 | 0.21 |
| PBI Mean Parental Care | Parental diff in Overvaluation | -0.03 | 0.02 | -2.23 | 0.026 | -0.12 |
| PBI Mean Parental Care | Mean Parental Care*Oval | -0.11 | 0.04 | -2.67 | 0.008 | -0.28 |
| PBI Mean Parental Care | Mean Parental Oval*Leni | -0.07 | 0.03 | -2.38 | 0.017 | -0.20 |
|  |  |  |  |  |  |  |
| PBI Parental diff in Care | PBI Mean Parental Leniency | 0.00 | 0.01 | 0.21 | 0.831 | 0.01 |
| PBI Parental diff in Care | PBI Parental diff in Leniency | 0.03 | 0.01 | 3.76 | 0.000 | 0.31 |
| PBI Parental diff in Care | PBI Mean Parental Overpro | 0.01 | 0.01 | 0.48 | 0.630 | 0.03 |
| PBI Parental diff in Care | PBI Parental diff in Overpro | -0.02 | 0.01 | -2.73 | 0.006 | -0.19 |
| PBI Parental diff in Care | Mean Parental Overvaluation | -0.02 | 0.02 | -0.84 | 0.404 | -0.04 |
| PBI Parental diff in Care | Parental diff in Overvaluation | 0.09 | 0.01 | 6.60 | 0.000 | 0.50 |
| PBI Parental diff in Care | Mean Parental Care*Oval | 0.01 | 0.01 | 0.39 | 0.700 | 0.02 |
| PBI Parental diff in Care | Mean Parental Oval*Leni | 0.01 | 0.01 | 0.43 | 0.665 | 0.03 |
| PBI Mean Parental Leniency | PBI Parental diff in Leniency | 0.00 | 0.01 | -0.03 | 0.973 | 0.00 |
| PBI Mean Parental Leniency | PBI Mean Parental Overpro | -0.18 | 0.02 | -8.66 | 0.000 | -0.65 |
| PBI Mean Parental Leniency | PBI Parental diff in Overpro | 0.00 | 0.01 | 0.15 | 0.880 | 0.01 |
| PBI Mean Parental Leniency | Mean Parental Overvaluation | 0.14 | 0.04 | 3.86 | 0.000 | 0.25 |
| PBI Mean Parental Leniency | Parental diff in Overvaluation | 0.01 | 0.02 | 0.56 | 0.576 | 0.03 |
| PBI Mean Parental Leniency | Mean Parental Care*Oval | -0.10 | 0.04 | -2.59 | 0.010 | -0.25 |
| PBI Mean Parental Leniency | Mean Parental Oval*Leni | -0.11 | 0.04 | -2.76 | 0.006 | -0.33 |
| PBI Parental diff in Leniency | PBI Mean Parental Overpro | -0.01 | 0.01 | -1.14 | 0.256 | -0.06 |
| PBI Parental diff in Leniency | PBI Parental diff in Overpro | -0.06 | 0.01 | -6.83 | 0.000 | -0.58 |
| PBI Parental diff in Leniency | Mean Parental Overvaluation | 0.02 | 0.02 | 1.01 | 0.313 | 0.06 |
| PBI Parental diff in Leniency | Parental diff in Overvaluation | 0.02 | 0.01 | 1.67 | 0.095 | 0.12 |
| PBI Parental diff in Leniency | Mean Parental Care*Oval | -0.02 | 0.02 | -1.01 | 0.312 | -0.07 |
| PBI Parental diff in Leniency | Mean Parental Oval*Leni | -0.01 | 0.01 | -0.69 | 0.491 | -0.04 |
| PBI Mean Parental Overpro | PBI Parental diff in Overpro | 0.02 | 0.01 | 2.05 | 0.040 | 0.11 |
| PBI Mean Parental Overpro | Mean Parental Overvaluation | -0.04 | 0.04 | -1.00 | 0.319 | -0.06 |
| PBI Mean Parental Overpro | Parental diff in Overvaluation | -0.01 | 0.02 | -0.59 | 0.555 | -0.03 |
| PBI Mean Parental Overpro | Mean Parental Care*Oval | 0.10 | 0.04 | 2.71 | 0.007 | 0.24 |
| PBI Mean Parental Overpro | Mean Parental Oval*Leni | 0.10 | 0.04 | 2.66 | 0.008 | 0.29 |
| PBI Parental diff in Overpro | Mean Parental Overvaluation | 0.01 | 0.02 | 0.43 | 0.667 | 0.03 |
| PBI Parental diff in Overpro | Parental diff in Overvaluation | 0.01 | 0.01 | 0.62 | 0.536 | 0.05 |
| PBI Parental diff in Overpro | Mean Parental Care*Oval | 0.01 | 0.02 | 0.71 | 0.479 | 0.05 |
| PBI Parental diff in Overpro | Mean Parental Oval*Leni | 0.01 | 0.01 | 0.56 | 0.576 | 0.04 |
| Mean Parental Overvaluation | Parental diff in Overvaluation | -0.05 | 0.03 | -1.70 | 0.089 | -0.09 |
| Mean Parental Overvaluation | Mean Parental Care*Oval | -0.14 | 0.09 | -1.56 | 0.118 | -0.16 |
| Mean Parental Overvaluation | Mean Parental Oval*Leni | -0.07 | 0.07 | -0.91 | 0.365 | -0.09 |
| Parental diff in Overvaluation | Mean Parental Care*Oval | 0.02 | 0.02 | 0.73 | 0.465 | 0.04 |
| Parental diff in Overvaluation | Mean Parental Oval*Leni | 0.02 | 0.02 | 1.02 | 0.306 | 0.05 |
| Mean Parental Care*Oval | Mean Parental Oval*Leni | 0.26 | 0.09 | 3.04 | 0.002 | 0.52 |

*References*

1. Weston R, Gore PA. A Brief Guide to Structural Equation Modeling. The Counseling Psychologist. 2006;34(5):719-51.

2. Rosseel Y. lavaan: An R Package for Structural Equation Modeling. Journal of Statistical Software. 2012;48(2):1-36.

3. Kenny DA, Kaniskan B, McCoach DB. The Performance of RMSEA in Models With Small Degrees of Freedom. Sociological Methods and Research. 2015;44(3):486-507.
